# C-reactive protein derived from perivascular adipose tissue accelerates injury-induced neointimal hyperplasia

**DOI:** 10.1186/s12967-020-02226-x

**Published:** 2020-02-11

**Authors:** Jia-Yuan Chen, Xiao-Lin Zhu, Wen-Hao Liu, Yong Xie, Hai-Feng Zhang, XiaoQiao Wang, Ru Ying, Zhi-Teng Chen, Mao-Xiong Wu, Qiong Qiu, Jing-Feng Wang, Yang-Xin Chen

**Affiliations:** 1 Department of Cardiology, Sun Yat-sen Memorial Hospital of Sun Yat-sen University, No. 107, Yanjiang West Road, Yuexiu District, Guangzhou, 510120 People’s Republic of China; 2 Department of Cardiology, The Second Affiliated Hospital of Guangzhou Medical University, Guangzhou, 510000 People’s Republic of China; 3 Department of Anesthesiology, The Second Affiliated Hospital of Guangzhou Medical University, Guangzhou, 510000 People’s Republic of China; 4 Department of Cardiology, The First Affiliated Hospital of NanChang University, Nanchang, 330006 People’s Republic of China

**Keywords:** C-reactive protein, Chemokine (C-X-C motif) ligand 7, Macrophage, Perivascular adipose tissue, Vascular injury

## Abstract

**Aim:**

Inflammation within the perivascular adipose tissue (PVAT) in obesity plays an important role in cardiovascular disorders. C-reactive protein (CRP) level in obesity patients is significantly increased and associated with the occurrence and progression of cardiovascular disease. We tested the hypothesis CRP derived from PVAT in obesity contributes to vascular remodeling after injury.

**Methods:**

A high-fat diet (HFD) significantly increased CRP expression in PVAT. We transplanted thoracic aortic PVAT from wild-type (WT) or transgenic CRP-expressing (CRPTG) mice to the injured femoral artery in WT mice.

**Results:**

At 4 weeks after femoral artery injury, the neointimal/media ratio was increased significantly in WT mice that received PVAT from CRPTG mice compared with that in WT mice that received WT PVAT. Transplanted CRPTG PVAT also significantly accelerated adventitial macrophage infiltration and vasa vasorum proliferation. It was revealed greater macrophage infiltration in CRPTG adipose tissue than in WT adipose tissue and CRP significantly increased the adhesion rate of monocytes through receptor Fcγ RI. Proteome profiling showed CRP over-expression promoted the expression of chemokine (C-X-C motif) ligand 7 (CXCL7) in adipose tissue, transwell assay showed CRP increased monocyte migration indirectly via the induction of CXCL7 expression in adipocytes.

**Conclusion:**

CRP derived from PVAT was significantly increased in HFD mice and promoted neointimal hyperplasia after vascular injury.

## Introduction

Obesity is a major risk factor for cardiovascular diseases and the incidence has risen substantially nowadays [1]. Obesity, accompanied by chronic low-grade inflammation in adipose tissue, is causally linked to the initiation and progression of multiple obesity-related vascular disorders, including atherosclerosis and post-PCI (percutaneous coronary intervention) restenosis [2–4]. Specially, inflammation within perivascular adipose tissue (PVAT) has emerged as a bridge to between vascular diseases and obesity due to its distinctive location and local effects, which is characterized by inflammatory cytokine secretion and inflammatory cell infiltration [5, 6]. Unlike that in other types of adipose tissue, the mechanisms involved in PVAT inflammation primarily involve the autocrine or paracrine pathway, and perivascular adipocytes possess a stronger ability to secrete a variety of inflammatory cytokines, such as monocyte chemoattractant protein-1 (MCP-1) and tumor necrosis factor-α (TNF-α), upon exposure to harmful stimuli [7]. Moreover, without fascia separating PVAT from the intima-media, the interplay between the arterial wall and its PVAT is bidirectional, not only vascular inflammation affects the pathophysiology of PVAT through paracrine signals, but also cytokines produced by adipocytes can permeate freely through the adventitia to the intima, and then attract immune cells from peripheral blood to the damaged intima, contributing to the pathogenesis of vascular disease [8, 9]. Experimental studies showed transplanted PVAT accelerated injury-induced neointimal hyperplasia in a mouse model and PVAT-derived leptin promoted neointima formation after balloon-induced injury in mice [10, 11]. As above mentioned, the local inflammatory cytokine deprived from PVAT is crucial to the pathogenesis of vascular disease, especially for patient with obesity. C-reactive protein (CRP) is a frequently used marker of systemic inflammation, the latest landmark CANTOS trial showed those with CRP concentrations less than 2 mg/L achieved by canakinumab treatment (a monoclonal antibody targeting interleukin 1β) had a 25% reduction of major adverse cardiovascular events, which indicated the prognostic value of CRP in primary and secondary prevention of cardiovascular diseases [12]. and elevation of the preprocedural CRP level in patients undergoing PCI was shown to be associated with the incidence of adverse cardiac events, such as: in-stent restenosis, especially for whose with obesity and diabetes [13–17]. Higher baseline hs-CRP level (≥ 4.08 mg/dL) in obese acute myocardiac infarction patients undergoing PCI showed significant association with 1-year all-cause mortality [18]. However, the underlying mechanism remains unknown. It is a generally accepted view that circulating CRP acts on the endothelium and the luminal side of the vessel wall to induce adhesion and migration of leukocytes and macrophages into the artery wall [19], which contributes to smooth muscle cell proliferation. On the other hand, little attention has been paid to the role of CRP produced in the adventitia and PVAT in response to injury, and whether CRP secreted by PVAT promotes neointima formation after vascular injury had not been determined.

Research has shown that in pathological conditions, adipose tissue can generate high levels of CRP, which plays an important role in the progression of many forms of chronic disease [20]. For example, Kaneko et al. showed that CRP expression is significantly increased in the subcutaneous adipose tissue of obese patients, which in turn aggravates glucose metabolism disorder and insulin resistance [21]. Peyrinbiroulet et al. reported that CRP produced by mesenteric visceral adipose tissue in patients with Crohn’s disease also is involved in the development of intestinal inflammation [22]. Our previous study demonstrated that delivery of exogenous CRP could induce arterial endothelial dysfunction by activating an inflammatory reaction within PVAT [23].

Based on these previous findings, we hypothesized that CRP derived from PVAT promotes neointima formation after vascular injury. Because exogenous human CRP preparations could be contaminated by bacterial endotoxin by-products [24], we established transgenic mice overexpressing human CRP (CRPTG mice) specifically in adipose tissue to avoid the confounding effects of contamination in CRP preparations. In addition, considering that the production of CRP requires coordination with androgen production in vivo [25], only male CRPTG mice were used. In the present study, we performed vascular endothelial wire injury model, for which intraluminal insertion of a large wire will lead to endothelia cell denuded and smooth muscle cell hyperplasia in response to transluminal mechanical injury, which resembles post-interventional vascular remodeling [26]. We investigated whether CRP derived from PVAT promotes neointimal hyperplasia after vascular injury, as well as the underlying molecular mechanism, with the goal of providing evidence for the potential of CRP as a novel target for preventing vascular restenosis after angioplasty in cardiovascular high-risk patient with obesity and diabetes.

## Materials and methods

### Animals and experimental protocol

#### Animal experiment 1

The animal experiment 1 and 2 were conducted according to the guidelines and ethical standards of the Animal Care and Use Ethics Committees of Sun Yat-Sen University (IACUC-DB-16-072). Eight-week-old male C57BL/6J mice and high-fat (HFD) feed (0.15% cholesterol, 21% lard, 78.85% basic feed, D12492) were purchased from Guangdong Province Medical Animal Center. The mice received a HFD for 2–8 weeks before the thoracic aortic PVAT was removed to examine the expression of CRP by polymerase chain reaction and western blot analyses.

#### Animal experiment 2

Transgenic mice expressing human CRP only in adipose tissue via the mouse Fabp4 promoter (specific expression in fat tissue) [27] were purchased from Cyagen Bioscience Inc (Guangzhou, China). Transgenic mice and wild-type (WT) littermates on a C57BL/6N background were used for the experiments performed in this study. To examine the effects of transplanted PVAT on injured arteries, neointimal hyperplasia in injured arteries was examined at 4 weeks after the vascular injury. The intimal and medial areas were measured, and the ratio of neointima/media area was calculated. Mice received a standard chow diet (STD).

### Femoral artery injury and adipose tissue transplantation

#### Femoral artery injury

As described in detail previously [23], the mice were anesthetized with a mixture of ketamine (8 mg/100 g) and xylazine (1.2 mg/100 g) administered intraperitoneally. The femoral artery, vein, and nerve bundle were exposed, and 6-0 silk sutures were placed in the proximal and distal ends of the femoral vascular bundle. The profunda femoris artery was isolated, and 6-0 silk sutures were placed beneath both ends of the artery before a loop was created at the distal end. The exposed profunda femoris artery was dilated by local application of one drop of 1% lidocaine hydrochloride. Transverse arterioctomy was performed in the profunda femoris artery with vannas scissors. A guide wire was inserted into the artery via the cut, and the femoral artery was stretched to reduce the angle between the profunda femoris artery and femoral artery. The wire was inserted into the proximal ligation along the long axis of the artery and was left in place for 2 min to denude the artery. Then the wire was removed, and the silk suture was looped at the proximal portion.

#### Adipose tissue transplantation

Mice were sacrificed, and after exposure of the thoracic aorta, the PVAT around the thoracic aorta was carefully removed with microforceps under a surgical microscope. The PVAT was placed in Dulbecco’s Modified Eagle Medium (DMEM, Gibco, Thermo Fisher Scientific, Inc., Waltham, MA, USA) with containing 1% antibiotics (R&D Systems, Inc., Minneapolis, MN, USA). The left femoral artery was denuded using a guide wire as described above, and then after removal of endogenous PVAT, the injured femoral artery was wrapped with transplanted PVAT.

### Quantification of neointimal hyperplasia

Four weeks after surgical intervention, the mice were killed by intraperitoneal administration of an overdose of nembutal, and then the mice were perfused via the left ventricle with 0.9% NaCl solution followed by 4% paraformaldehyde in phosphate-buffered saline (PBS, pH 7.4). The femoral artery and transplanted PVAT were carefully excised, postfixed in 4% paraformaldehyde overnight at 4 °C, and embedded in paraffin. Cross-sections (5 μm) were cut and then stained with hematoxylin and eosin (H&E), and the neointimal and medial areas of the artery were measured utilizing image ImageJ 1.48 software (National Institutes of Health).

### Immunohistochemical and immunofluorescene analyses

Paraffin-embedded sections of the femoral artery and transplanted PVAT (5 μm thick) were deparaffinized and blocked with 0.5% horse serum. The sections were then incubated with primary antibody against human C-reactive protein (Santa Cruz Biotechnology, Inc., Santa Cruz, California, USA), CD68 (Santa Cruz Biotechnology, Inc., Santa Cruz, California, USA), CD31 (R&D Systems, Inc., Minneapolis, MN, USA) or isotype-specific antibody, followed by incubation with goat anti-mouse immunoglobulin (Ig) [F(abʹ)2] conjugated with peroxidase (Amersham Pharmacia Biotech Inc, Piscataway, NJ, USA) as the secondary antibody at room temperature for 60 min. The sections were then counterstained with hematoxylin. Peroxidase activity was visualized by incubation with a 3,3-diaminobenzidine solution and observed under light microscopy. The quantity of CD68+ in adventitia around injured femoral artery was determined according to a previously reported method with minor modification [28]. The quantity of adventitial macrophages was counted manually the number of CD68-related antigen positive cell at 400 times magnification by two independent observers.

For immunofluorescene analysis, Paraffin-embedded sections of perivascular adipose tissue (5 μm thick) were deparaffinized and blocked with 0.5% horse serum. After permeabilisation, sections were incubated with primary a antibodies over night at 4 °C. The antigen was detected with an anti-rabbit Alexa488 (green) and anti-mouse Alexa555 (red) conjugated secondary antibody (1/500). DAPI (blue) was used for nuclei staining. After permeabilisation, cells were incubated with antibodies to rabbit anti-TIP47 (perilipin 3/TIP47,adipocytes marker, Abcam Inc, Abcam, CA, USA) and mouse anti-CD68 (macrophage marker, Santa Cruz Biotechnology, Inc., Santa Cruz, California, USA). Antigen was detected with fluorescently labeled secondary antibodies as described above. Specimens were analyzed by confocal microscopy.

### Flow cytometric analysis

After the animals were sacrificed, the perivascular and subcutaneous adipose tissues were carefully removed and completely immersed in D-Hanks buffer containing 100 U/mL penicillin and 0.1 mg/mL streptomycin. After the removal of visible blood vessels, lymph nodes, and fascia, the tissue was finely minced with scissors and digested with collagenase type I (1.25% w/v, Invitrogen, Thermo Fisher Scientific, Inc., Waltham, MA, USA) for 60 min at 37 °C with gentle shaking. After neutralization of the collagenase, the floating adipocytes were separated by centrifugation at 1200 rpm for 5 min. The resulting pellet of vascular stromal cell components was resuspended in PBS and filtered through a 70-μm cytoscreener. Finally, the cell suspensions were probed with mouse anti-CD11b-FITC antibody (Invitrogen, Thermo Fisher Scientific, Inc., Waltham, MA, USA) via incubation for 20 min in darkness, and the expression of CD11b was detected by FACS Aria cell sorting system (LSRII FACS; BD Bioscience, Franklin Lakes, NJ, USA) and results were analyzed by Flowjo10 software.

### Gel-free quantitative proteomic profiling in adipose tissue of CRPTG mice

#### Protein extraction

After the animals were sacrificed, the perivascular adipose tissues were carefully removed and grinded to powder in liquid nitrogen, and the blood samples were collected. Proteins were extracted in lysis buffer (8.4 M urea, 2.4 M thiourea, 5% CHAPS, 50 mM DTT, and 1% IPG buffer) for 30 min on ice, and then cells were further broken using an ultrasonic cell disruptor, followed by centrifugation at 14,000 rpm for 1.5 h at 19 °C using a TL-100 ultracentrifuge (Beckman, Palo Alto, CA, USA). Finally, the middle layer of aqueous liquid was retained.

1. *Trypsin digestion and labeling of adipose tissue samples with TMT* Protein pellets were suspended in 100 mL of 200 mM TEAB and digested overnight at 37 °C with 2.5 μg sequencing grade modified trypsin (Promega, Madison, WI, USA). Six digested samples were individually labeled with TMT6 reagents according to the manufacturer’s instructions. Three littermate control samples and three CRPTG mouse samples were used in this experiment.
2. *Liquid chromatography tandem mass spectrometry (LC*–*MS/MS) analysis and database searches* Mass spectrometric analysis of the TMT-labeled samples was performed on an Ultimate 3000 Dionex LC system (Dionex, Sunnyvale, CA, USA) connected to a Q Exactive mass spectrometer (Thermo Fisher Scientific, Inc., Waltham, MA, USA) operated according to a higher energy collisional dissociation model. The detailed steps were described previously [29]. Protein identification and quantification based on LC–MS/MS data were performed with Proteome Discoverer 1.4 (Thermo Fisher Scientific, Inc., Waltham, MA, USA) interfaced with Uniprot (mouse_81798_20161104.fasta).

### Isolation and purified of CD14+ human monocytes

The isolation of human monocytes were conducted according to ethical standards of Ethics Boards of Sun Yat-Sen Memorial Hospital of Sun Yat-sen University (EBSYSM-16-022). Heparinized peripheral blood obtained from healthy volunteers was mixed with PBS. After being let stand for 1 h at room temperature, the upper leukocyte-enriched plasma layer was harvested and centrifuged, after which the cell pellet was resuspended in PBS and placed onto a Ficoll (ICN Biomedicals, Irvine, CA, USA) and centrifuged at 700*g* for 30 min. Red blood cells and polymorphonuclear leukocytes (PMN) are dense and centrifuge through the medium while peripheral blood mononuclear cells (PBMCs; mainly monocytes and lymphocytes) band over Ficoll and can be recovered at the interface. The recovered mononuclear cells were washed three times with PBS buffer. Isolation of CD14+ monocytes was performed using magnetic bead-based separation with CD14 MicroBeads, the MiniMACS separator and LS type columns (Miltenyi Biotec, Germany). Purified CD14+ monocytes were resuspended in RPMI 1640 medium containing 10% FBS (both form Invitrogen, Thermo Fisher Scientific, Inc., Waltham, MA, USA). The purity of CD14+ isolation was > 90%, as determined by flow cytometry.

### Cell migration assay

Cell migration experiment was performed using a co-culture system. Primary human peripheral blood mononuclear cells (PBMCs) were seeded on the upper side of 8.0-µm Transwell membrane plates (Corning, Inc., NY, USA) at a density of 5 × 10^4^ cells/well after serum starvation for 12 h. Culture medium alone (control), 3T3-L1 adipocyte conditioned medium (CM) only, CM of 3T3-L1 adipocyte treated with CRP (free of sodium azide; Sino Biological Inc., Beijing, China), or CM of 3T3-L1 adipocyte treated with CRP plus anti-CXCL7 blocking antibody (R&D Systems, Inc., Minneapolis, MN, USA) or isotype-match IgG was introduced in the lower wells of the transwell membrane plates for 12 h. Migrating cells remaining on the transwell membrane were fixed, then stained using 10% crystal-violet (Sigma-Aldrich, St. Louis, MO, USA), and counted under a light microscope.

### Adhesion test

As described in detail previously [30], recombinant human CRP (free of sodium azide; Sino Biological Inc. Beijing, China) at different concentrations was mixed with 10% poly-lysine (FN), and 200 μL of the suspension was added to the wells of a 96-well plate for incubation at 4 °C overnight. The wells were then blocked by incubation of 3% bovine serum albumin in PBS at 37 °C for 1 h. The concentrations of primary human PBMCs and THP-1 cells (ATCC, Manassas, USA) were adjusted to 1 × 10^5^ cells/mL with serum-free medium. THP-1 cells were treated with 200 nM phorbol ester (PMA) with or without anti-CD16/32 or anti-CD64 neutralizing antibody (R&D Systems, Inc., Minneapolis, MN, USA) at 37 °C for 1 h before being seeded in a 96-well plate coated with CRP and allowed to incubate at 37 °C for 1 h. Then non-adherent cells were washed away with PBS, and adherent cells remaining on the plate were fixed and stained using 10% crystal-violet. The cells remaining on the plate were counted under light microscopy.

### Enzyme-linked immunosorbent assay for CXCL 7

3T3-L1 preadipocytes (obtained from ATCC) were maintained in DMEM containing 10% FCS. Adipocyte differentiation was initiated by adding differentiation medium that contained 10 μg/mL insulin, 0.25 μM dexamethasone, and 0.5 mM 3-isobutyl-1-methylxanthine. After 48 h, the medium was replaced with medium containing 10 μg/mL insulin, and cells were maintained in this medium until use. Fully differentiated 3T3-L1 cells were starved for 12 h, and then were stimulated with different concentrations of exogenous CRP recombinant protein. The supernatants were collected 24 h later. The concentration of CXCL7 in cell supernatants and plasma sample from mice was examined by enzyme-linked immunosorbent assay (ELISA) using a commercially available kit (Raybiotech, Atlanta, GA, USA) according to the manufacturer’s instructions.

### Western blot analysis

For preparation of the protein extracts, the cells were rinsed twice with ice-cold PBS, centrifuged, and resuspended in lysis buffer (Thermo Fisher Scientific, Waltham, MA, USA) for 30 min on ice. Protein concentrations were assayed using Coomassie Plus reagent (Pierce) according to the manufacturer’s instructions, and 40 or 100 µg of protein was loaded for separation by sodium dodecyl sulfate-polyacrylamide gel electrophoresis (SDS-PAGE). The proteins were then transferred to polyvinylidene difluoride membranes (Immobilon-P; EMD Millipore Corporation, Billerica, MA, USA). The membranes were blocked in Tris-buffered saline containing 5% BSA and probed with CXCL7, TNF-ɑ and MCP-1antibodies (all purchased from R&D Systems, Inc., Minneapolis, MN, USA) Protein bands were detected by horseradish peroxidase-conjugated secondary antibodies and enhanced chemiluminescence substrates (PerkinElmer, Boston, MA, USA).

### Quantitative real-time polymerase chain reaction analysis

Total RNA was extracted using Trizol reagent (Invitrogen, Thermo Fisher Scientific, Inc., Waltham, MA, USA). cDNA was synthesized on DNaseI-treated total RNA templates (0.5 μg) using an iscriptTMcDNA synthesis kit (Takara Bio, Inc., Shiga, Japan). Gene expression was assessed by quantitative real-time polymerase chain reaction (QPCR) using SYBR Green intercalating dye (Invitrogen, Thermo Fisher Scientific, Inc., Waltham, MA, USA) and mouse primers. The primer sequences for mouse CRP were: sense: 5′- TTCCCAAGGAGTCAGATACTTCC-3′, and antisense: 5′-TCAGAGCAGTGTAGAAATGGAGA-3′. The comparative threshold cycle method was used to calculate the fold amplification as specified by the manufacturer. The amplified PCR products were separated by gel electrophoresis in a 2% agarose gel and visualized with ethidium bromide. Each sample was replicated at least three times.

### Statistical analysis

The in vitro data are representative of independent experiments performed in triplicate. The statistical analysis was conducted using SPSS 16.0 software (SPSS, Inc., Chicago, IL, USA). The statistical significance of the differences among groups was tested using one-way analysis of variance or the Mann–Whitney test, multiple comparison between the groups was performed using S-N-K method and *P *< 0.05 was considered indicative of a significant difference. Error bars are indicative of standard error of mean.

## Results

### Effects of HFD on CRP expression in PVAT in mice and confirmation of adipose tissue-specific over-expression of human CRP in CRPTG mice

**Fig. 1:**
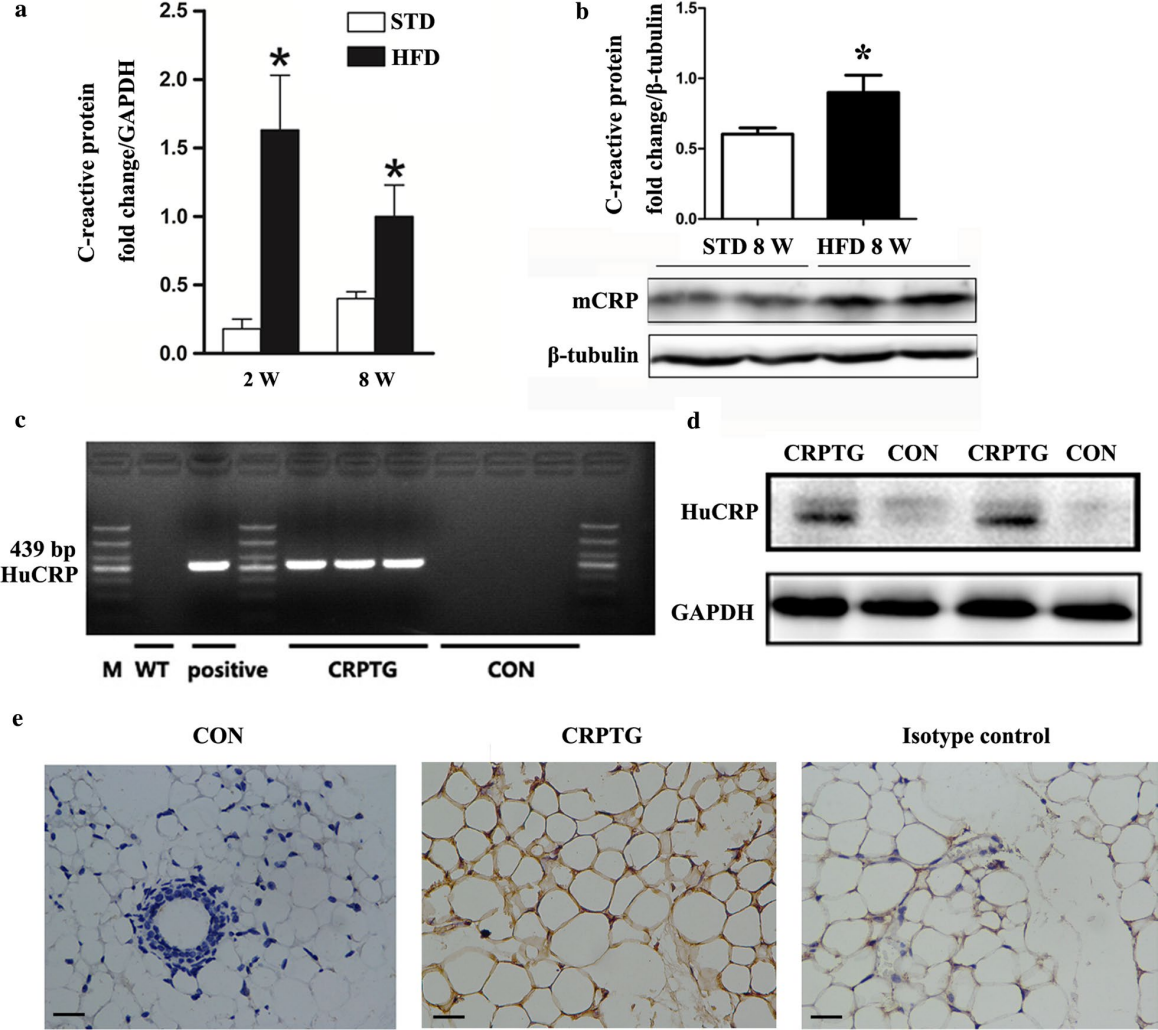
Effect of HFD on the expression of CRP in PVAT and confirmation of CRP expression specifically in adipose tissue of CRPTG mice. **a** mRNA expression of CRP in PVAT was quantified by RT-PCR after WT C57BL6 mice received the HFD for 2 or 8 weeks. **b** Protein expression of CRP in PVAT was quantified by western blotting after mice were fed the HFD for 8 weeks; the values were normalized to β-tubulin expression as a control. **c** Gene expression of human CRP was detected by PCR and agarose gel electrophoresis based on genomic DNA extracted from tails of TG mice. **d**, **e** Protein expression of human CRP in perivascular adipose tissue (PVAT) was detected by western blotting and immunohistochemical staining. *CON* control, *CRPTG* CRP-overexpressing transgenic mice, *huCRP* human C-reactive protein. Scale bars, 100 μm. *P < 0.05 versus standard diet. Data represent mean ± standard error of the mean (SEM, n = 3)

The body weight of the HFD group was significantly higher than that of the STD group after 8 weeks (25.9 ± 0.62 g vs. 34.5 ± 0.83 g, P = 0. 28, n = 8), and the HFD significantly increased the mRNA transcription and protein expression of CRP in PVAT (Fig. 1a, b). In addition, the gene and protein expression of human CRP was confirmed in F0 CRPTG mice and their offspring (Fig. 1c–e).

### Overexpression CRP in PVAT promoted neointimal hyperplasia after vascular injury

**Fig. 2:**
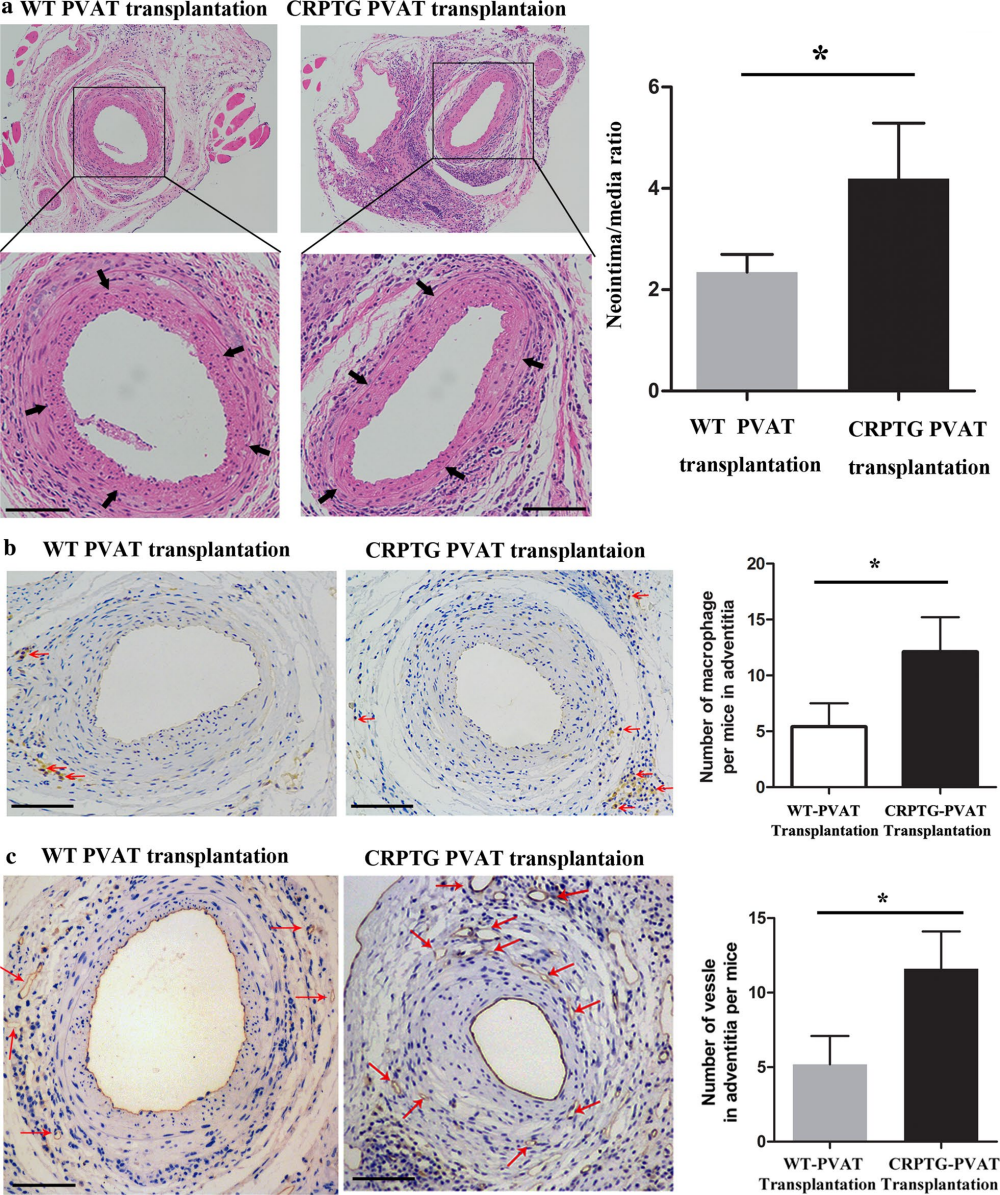
Overexpression of CRP in PVAT promoted neointimal hyperplasia, adventitial macrophage infiltration, and vasa vasorum proliferation at 4 weeks after endovascular injury. **a** Representative H&E-stained sections of femoral arteries after endovascular injury in STD-fed mice transplanted with WT PVAT or CRPTG PVAT. Arrows indicate internal elastic lamina. **b** CD68 (macrophage marker) staining revealed transplanted CRPTG PVAT markedly increased the numbers of adventitial macrophages surrounding injured arteries. Red arrows indicate CD68+ cells. **c** CD31 (endothelial cell marker, representative of vessels) staining showed transplanted CRPTG PVAT significantly increased adventitial adjacent to the injured vessel. Red arrows indicate CD31+ cells. Scale bars, 100 μm, *P < 0.05 versus WT PVAT transplantation. Data represent mean ± SE (n = 6)

The transplanted WT PVAT and CRPTG PVAT have the same survival rates in WT mice (75% vs. 75%). The neointima/media area ratio at 4 weeks after injury was significantly greater in WT mice transplanted with CRPTG PVAT compared to that in WT mice transplanted with WT PVAT (2.4 ± 0.35 vs. 4.4 ± 1.1, P = 0.0122, Fig. 2a). In addition, there were no significant difference in neointima thickness between transplanted with CRPTG PVAT and wild-type PVAT without wire injury (Additional file 1: Figure S1). Liver is known to contribute to CRP production, in order to demonstrate the role of CRP production in the liver under present experimental conditions, the detection of plama mouse CRP concentration in both groups was performed, we found there were no significant difference between two groups (Additional file 1: Figure S2), indicated the circulating CPR derived from liver is of little important in neointimal hyperplasia after vascular injury, as noted, the result should be interpreted cautiously owing to the small numbers.

### Transplanted CRPTG PVAT significantly accelerated adventitial macrophage infiltration and vasa vasorum proliferation after vascular injury

Staining for CD68 demonstrated increased macrophage infiltration in the adventitia after CRPTG PVAT transplantation compared with WT PVAT transplantation (*P *= 0.0142; Fig. 2b). Moreover, compared with that in WT mice transplanted with WT PVAT, the number of adventitia vasa vasorum was significantly greater in WT mice transplanted with CRPTG PVAT (*P *= 0.0321; Fig. 2c).

### Macrophage infiltration was enhanced in CRPTG adipose tissue in situ

**Fig. 3:**
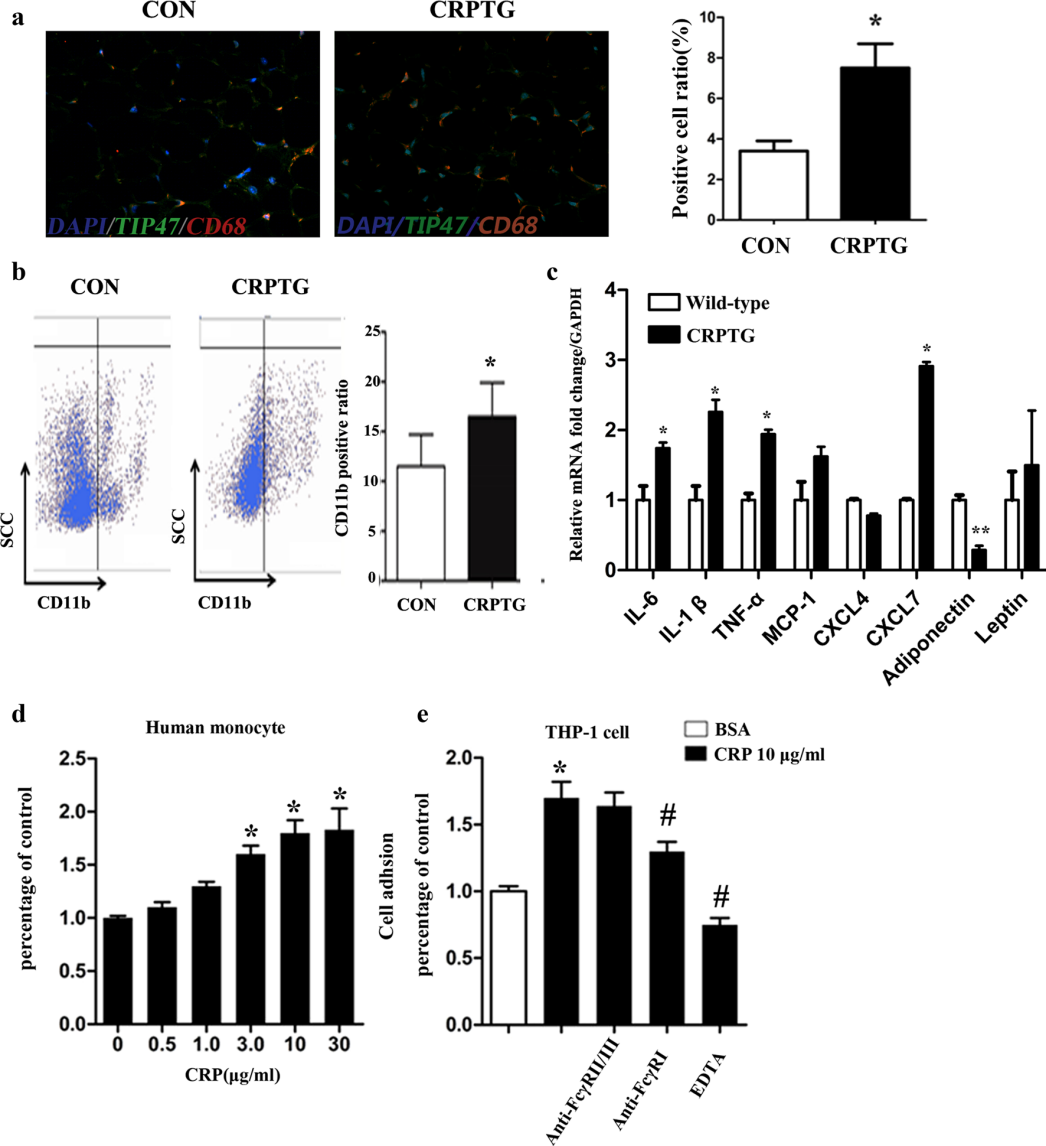
Macrophage infiltration was enhanced in CRPTG adipose tissue in situ and the effect of CRP on the adhesion of macrophage. **a** Immunohistochemical staining of adipose tissue from WT and CRPTG mice for the macrophage marker CD68 and adipocyte marker TIP47. Representative images and quantitative comparisons of CD68+ cells (n = 6) are shown. Scale bar, 100 μm. **b** Representative flow cytometric analysis of CD11b + macrophages in vascular stromal fraction isolated from adipose tissue. **c** Comparison of the expression of inflammatory cytokines between wild-type and CRPTG in perivascular adipose tissue examined by QPCR *P < 0.05 versus wild-type mice. **d** The effect of recombinant CRP on the adhesion rate of human PMBCs. *P < 0.05 versus 0 μg/mL CRP. **e** The effect of CRP on the adhesion rate of THP-1 cell-derived macrophages. *P < 0.05 versus BSA control. ^#^P < 0.05 versus 10 μg/mL CRP. Data represent mean ± SEM (n = 6)

Immunofluorescence staining showed that compared with the percentage in PVAT of non-transgenic littermates, significantly more CD68 + macrophages were observed in CRPTG PVAT (3.5 ± 0.3% vs. 7.6 ± 0.5%, *P *= 0.0154; Fig. 3a). Flow cytometric analysis further indicated that in the same mass of adipose tissue, the percentage of CD11b + macrophages in CRPTG was significantly greater by 4.9% compared with that in the littermate control group (*P *= 0.0402; Fig. 3b). QPCR showed C-reactive protein up-regulated the mRNA transcription level of inflammatory cytokines such as interleukin 1β (IL-1β), interleukin 6 (IL-6), tumor necrosis factor ɑ (TNF-ɑ) and neutrophil chemokine (C-X-C motif) ligand 7 (CXCL7) in CRPTG PVAT (Fig. 3c). The cell adhesion experiment showed that CRP significantly increased the adhesion rate of human PBMCs and THP-1 cell–derived macrophages in a concentration-dependent manner, and this effect was mainly mediated by CD64 (Fcγ RI) (Fig. 3d, e).

### Proteome profiling and western blot analyses showed overexpression of CRP promoted CXCL7 expression in adipose tissue

**Fig. 4:**
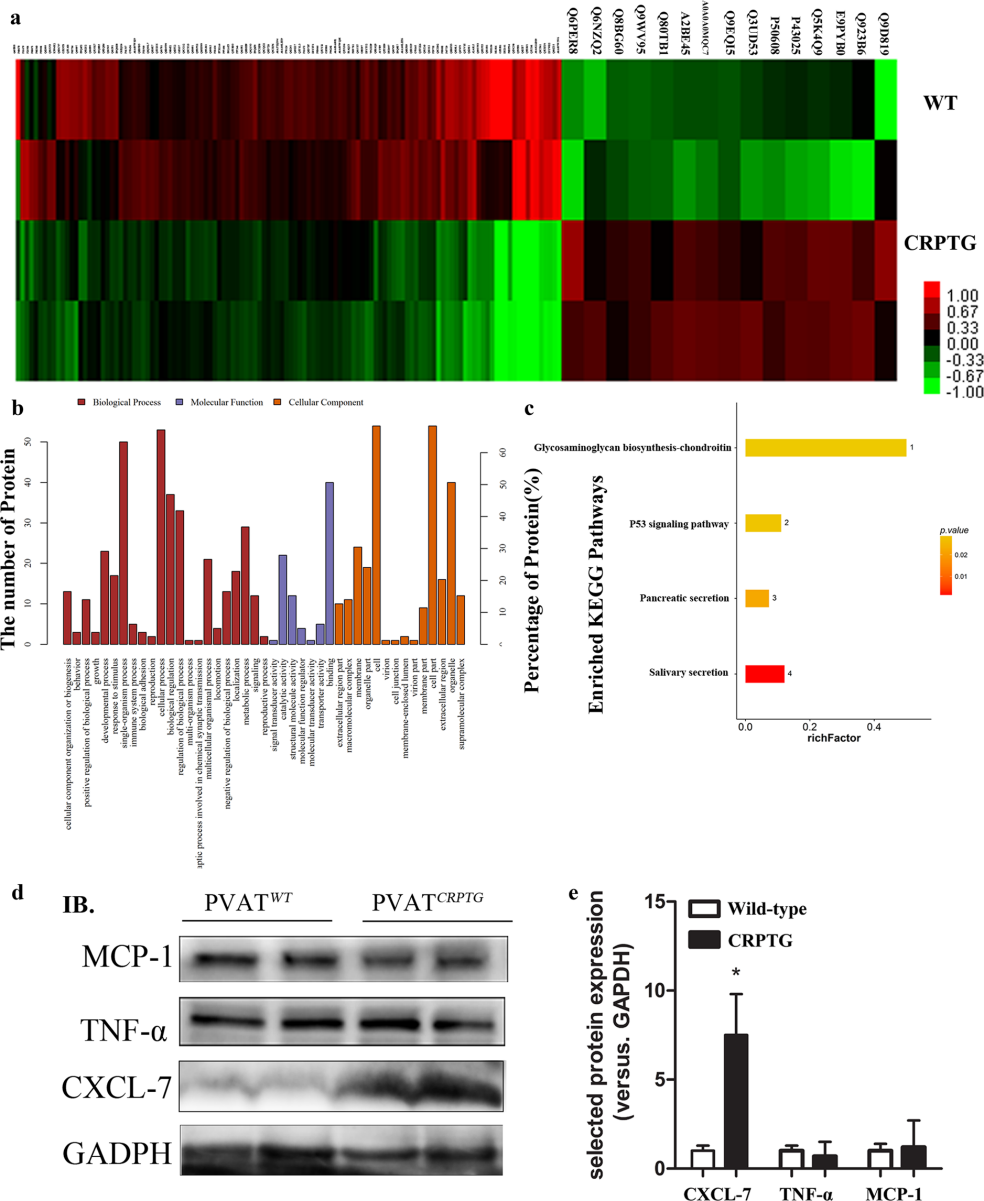
Quantitative proteome profiling of adipose tissue from WT and CRPTG mice and bioinformatics analysis of differentially expressed proteins. **a** Heat map analysis of the WT and CRPTG adipose tissue protein expression profiles, which showed that 137 differentially expressed proteins were detected. **b** BPGO (Biological Process and Gene Ontology) enrichment analysis of differentially expressed proteins between WT and CRPTG adipose tissues are involved organism processes, response to stimulus, cellular processes, biological regulation and metabolic processes. **c** Distribution of KEGG pathways. **d**, **e** CXCL-7 protein expression detected by quantitative western blot analysis. IB: immunity bolt. GAPDH was used as the loading control. *P < 0.05 versus WT-PVAT. Data represent mean ± SEM (n = 3)

In our LC–MS/MS analysis, all 5315 proteins identified by MS were subsequently classified via bioinformatics analysis (Additional file 2). Fifteen proteins were up-regulated, and 122 proteins were down-regulated in the CRPTG adipose tissue (Fig. 4a). Among these, CXCL7, a potent chemotactic cytokine, was increased significantly. To validate our proteomics data, western blot analyses were performed for selected proteins. As expected, western blot analyses showed that the expression level of CXCL7 protein was significantly greater in CRPTG adipose tissue than in WT adipose tissue, and there were no significant differences in the expression of TNF-ɑ and MCP-1 (Fig. 4d–e). Meanwhile, the circulating CXCL7 concentration of CRPTG mice was also significantly higher than that of WT mice examined by ELISA (19.0 ± 4.6 pg/mL vs. 8.1 ± 0.6 pg/mL, P = 0.0260, Additional file 1: Figure S3). BPGO (Biological Process and Gene Ontology) analysis showed that the differentially expressed proteins were predominantly involved in single organism processes, response to stimulus, cellular processes, biological regulation, metabolic processes, etc. (Figure 4b). KEGG (Kyoto Encyclopedia of Genes and Genomes) analysis showed that differentially expressed proteins were involved in the P53 signaling pathway (Fig. 4c).

### CRP promoted monocyte migration via induction of CXCL7 production in adipocytes

**Fig. 5:**
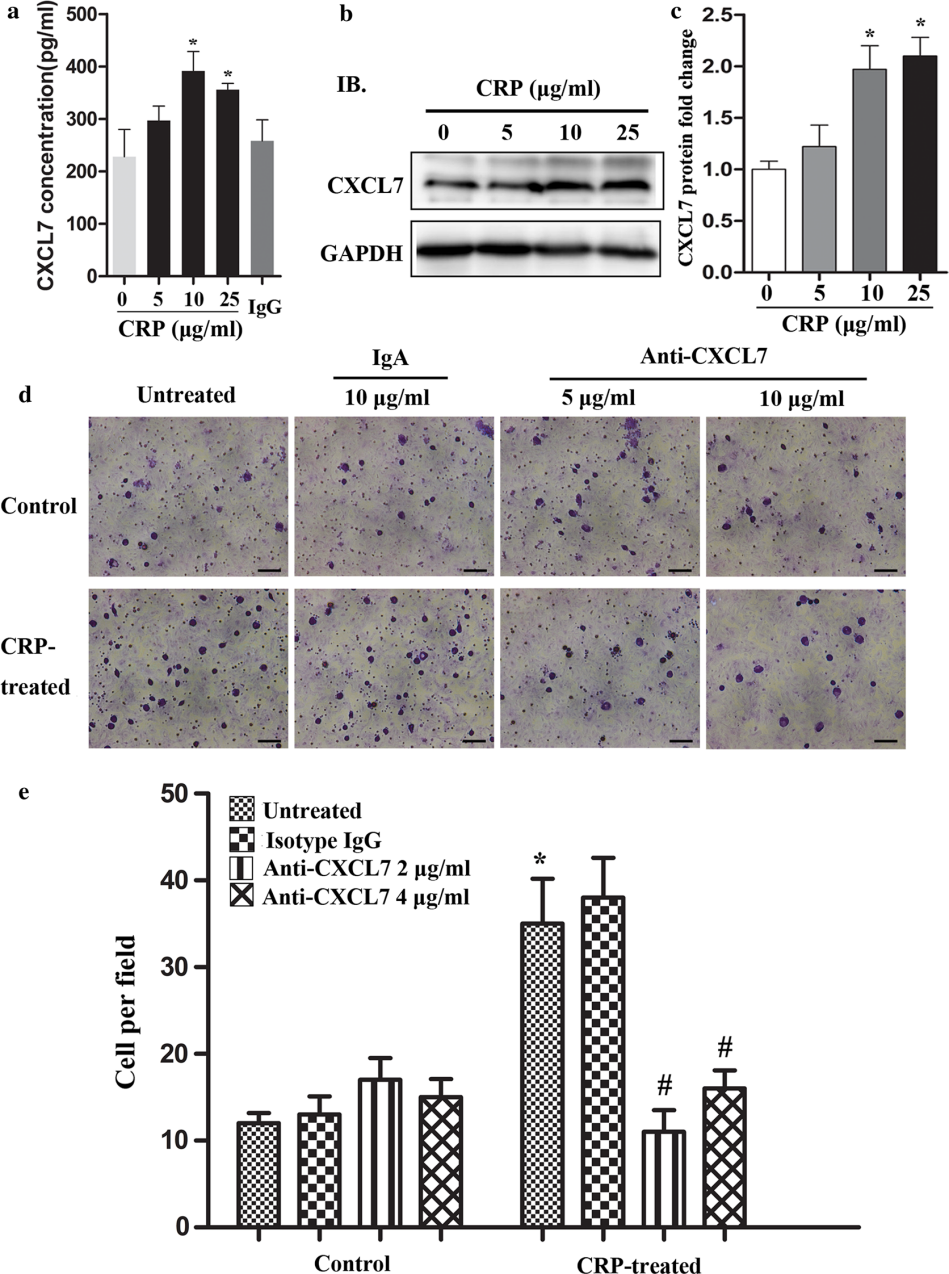
CRP promoted monocyte migration by inducing CXCL7 expression in adipocytes. **a** After 12 h of starvation, 3T3-L1 adipocytes were stimulated with different concentrations of exogenous CRP recombinant protein. The supernatants were collected 24 h later. CXCL7 production was determined by ELISA and it revealed that CRP dose-dependently increased CXCL7 production in 3T3-L1 adipocytes, with a peak CRP dose of 10 μg/mL. *P < 0.05 versus control. **b**, **c** CRP increased CXCL7 protein expression as assessed by western blotting. The values were normalized to β-tubulin expression as a control. *P < 0.05 versus control. **d**, **e** Transwell assays with primary human PBMCs plated on the upper cell culture inserts and culture medium alone (control), CRP-treated 3T3-L1 adipocytes plated in the lower chambers in the presence or absence of an anti-CXCL7 antibody at 2 or 4 μg/mL or an isotype-matched IgG control (IgG). Scale bar, 50 μm. *P < 0.05 compared with control (untreated), ^#^P < 0.05 compared with CRP-treated (untreated). Data represent mean ± SEM (n = 6)

Western blotting and ELISA analyses showed that CRP increased the synthesis and production of CXCL7 in 3T3-L1 adipocytes (Fig. 5a–c). In the presence of CRP-treated 3T3-L1 adipocytes, the number of migrated monocytes in the upper cell culture chamber was reduced upon addition of 2 or 4 μg/mL anti-CXCL7 antibody, but not by addition of 4 μg/mL isotype-matched IgG. Moreover, anti-CXCL7 antibody had no influence on the migration of monocytes in culture medium alone, indicating that CRP-treated CM from 3T3-L1 adipocytes induced primary human PBMC migration, and CXCL7 neutralization antibody could partially abrogate the CRP-mediated effect on monocyte migration (Fig. 5d, e).

## Discussion

CRP is the most evidence-based inflammatory factor in the field of cardiovascular diseases, but some clinical and epidemiological studies showed that CRP was not a causal factor by investigating the relationship between atherosclerosis and the changes of circulating CRP [31], however, these associations were underpowered after adjustment for established risk factors. Recently it is proposed that the effect of local microenvironment on the pathogenesis of atherosclerosis is important and the local inflammatory model should be taken in vivo experiments in order to avoid the cofounders caused by systemic inflammation [32]. The results of the present study showed CRP produced in PVAT significantly promoted neointimal hyperplasia with accelerating adventitial macrophage infiltration and vasa vasorum proliferation after endovascular injury. Furthermore, CRP itself was shown to directly enhance macrophage infiltration in adipose tissue and also to indirectly promote monocyte migration by inducing CXCL7 expression in adipocytes. Many prospective epidemiological studies have shown associations of circulating inflammatory markers with risk of cardiovascular disease (CVD), but these associations are modest after adjustment for confounders for the reason that systemic inflammatory markers are affected by a number of factors, however, to study the role of local inflammation environment in CVD could be even more crucial. Recent studies showed local inflammation within PVAT was an important risk factor for progression of vascular diseases. In physiology condition, functional PVAT protects the vessels by excreting vasodilator or thermogenic factors, but when PVAT is exposed to harmful stressors, such as obesity, diabetes, malnutrition and hypoxia, the adipose tissue changes from energy storage to a secretory phenotype, which is characterized by high levels of inflammatory cytokines, adipokines, and extracellular matrix metalloproteinases [33]. Previous researches have demonstrated that several molecules with possible autocrine or paracrine effects were produced by PVAT or epicardial adipose tissue in obesity, such as leptin, interleukin (IL)-6 and TNF-α, the expression of these molecules has been verified as independent risk factors in the pathogenesis of atherosclerosis [34, 35]. Our initial results showed that feeding a HFD increased the expression of CRP in PVAT, and in return, CRP deprived form PVAT promoted neointimal hyperplasia after endovascular injury, indicating that CRP derived from PVAT affects vascular remodeling after angioplasty and might represent a risk factor for post-PCI restenosis.

Adipose tissue is a heterogeneous organ that consists of mature adipocytes and stromal vascular cells, which includes mesenchymal stem/progenitor cells, endothelial cells, macrophages, and lymphocytes. Macrophage infiltration is an important feature of PVAT inflammation [36, 37]. Lohmann et al. showed macrophages were recruited to adventitial and PVAT in the atherosclerosis model of ApoE−/− mice, and the number of macrophages was positively correlated with arterial plaque size [38]. Furthermore, M1-like macrophages were found to promote plaque instability by degrading the extracellular matrix and inhibiting smooth muscle cell growth [39]. Therefore, inhibiting macrophage recruitment into PVAT may represent a new treatment option for cardiovascular diseases. The present study showed that transplanted CRPTG PVAT significantly accelerated adventitial macrophage infiltration in injured arteries and further confirmed that CRP enhanced macrophage infiltration in CRPTG adipose tissue ex vivo. These findings indicate that CRP produced by PVAT is also a potent chemotactic factor for monocytes, which in return aggravates the inflammatory response in PVAT. Overall, our data indicated that CRP might be a therapeutic target for PVAT inflammation by preventing macrophage recruitment.

Whether CRP acts as a strong pro-inflammatory cytokine remains controversial [40–42]. One of the most important reasons is that CRPpreparations in vitro experiment were reported to be contaminated by bacterial products or other contaminated preparations [43]. In present study, we generated transgenic mice expressing human CRP (CRPTG) in adipose tissue and compared the expression of inflammatory factors in PVAT between CRPTG and CON without contaminated confounders, we found CRP up-regulated the expression of inflammatory factors. To illustrate the signaling pathway and biological change involved in CRP-mediated effect in PVAT, an LC–MS/MS quantitative proteomics approach was performed. We identified 15 up-regulated proteins and 122 down-regulated proteins. Specially, CRP significantly increased the release of CXCL7 in adipose tissue. KEGG analysis showed several of these proteins were closely associated with pancreatic secretion, Paracrine or autocrine plays an crucial role in perivascular adipose tissue inflammation phenotypic transition. Among these, the CXCL7 protein level was significantly increased. CXCL7 was showed to involve in macrophages recruitment [44, 45]. However there was also negative result showed CXCL7failed to induce M1/2 macrophage chemotaxis, which could be attributable to the different state of the macrophage [46], In vitro experiments further showed that CRP promoted the monocyte migration by stimulating the production of CXCL7 by 3T3-L1 adipocytes, Besides, KEGG analysis also showed the P53 signal pathways activation in adipose tissue, It is reported p53 induces adipose tissue inflammation by promoting the development of insulin resistance. Our study showed CRP maybe exerts its inflammatory effect through activating p53 signaling pathway, but the specific mechanism needs further study.

### Limitations

The present study has several limitations that should be considered in the interpretation of the results. First, it is difficult to define the exact role of CRP in neointimal response to injury without CRP loss-of function studies, because CRP itself is expressed at very low level in mice. Second, we fail to compare the effects of transplanting adipose tissues from different locations, such as subcutaneous, perivascular and perirenal adipose tissue, experiments are need to further confirm the unique role of PVAT in the response to arterial injury. Third, it could be more informative to observe the effect of CRP on the adhesion of macrophages in CRPTG; CCR2-KO mice or by clodronate liposomes in vivo. Future studies should also include the use of CRPTG/CXCL7-/- double transgenic mice to test whether knockdown of CXCL7 expression can reverse the effects of CRP on macrophage infiltration.

Finally for only male mice were included, it should be interpreted cautiously when translating the research findings to clinical practice.

In conclusion, CRP derived from PVAT was significantly increased in obesity and promoted neointimal hyperplasia after vascular injury, partially through CRP-mediated enhancement of adventitial macrophage infiltration. The results of the present study indicate that CRP-derived PVAT may play a role in the pathogenesis of neointimal hyperplasia after angioplasty in obesity.

## Supplementary information

**Additional file 1: Figure S1.** Comparison of neointima thickness in the presence of wild-type and CRPTG PVAT transplantation without wire injury. **Figure S2.** Comparison of plama mouse C-reactive protein in WT mice received wild-type and CRPTG PVAT transplantation. **Figure S3.** Comparison of plama mouse CXCL-7 in CRPTG and wild-type mice.

**Additional file 2.** Original proteome profiling significant analysis data.

## Data Availability

Not applicable.

## Abbreviations

PVAT: inflammation within the perivascular adipose tissue
CRP: C-reactive protein
HFD: high-fat diet
CRPTG: transgenic CRP-expressing
CXCL7: chemokine (C-X-C motif) ligand 7
PCI: percutaneous coronary intervention)
MCP-1: monocyte chemoattractant protein-1
TNF-α: tumor necrosis factor-α
STD: standard chow diet
WT: wild-type
