# Supplementary material for: C-reactive protein derived from perivascular adipose tissue accelerates injury-induced neointimal hyperplasia
Source: J Transl Med. 2020 Feb 11;18:68. doi: 10.1186/s12967-020-02226-x (PMC7011279; doi:10.1186/s12967-020-02226-x)
Supplement: Supplementary file 1 — Additional file 1: Figure S1. Comparison of neointima thickness in the presence of wild-type and CRPTG PVAT transplantation without wire injury. Figure S2. Comparison of plama mouse C-reactive protein in WT mice received wild-type and CRPTG PVAT transplantation. Figure S3. Comparison of plama mouse CXCL-7 in CRPTG and wild-type mice. [file 12967_2020_2226_MOESM1_ESM.docx]

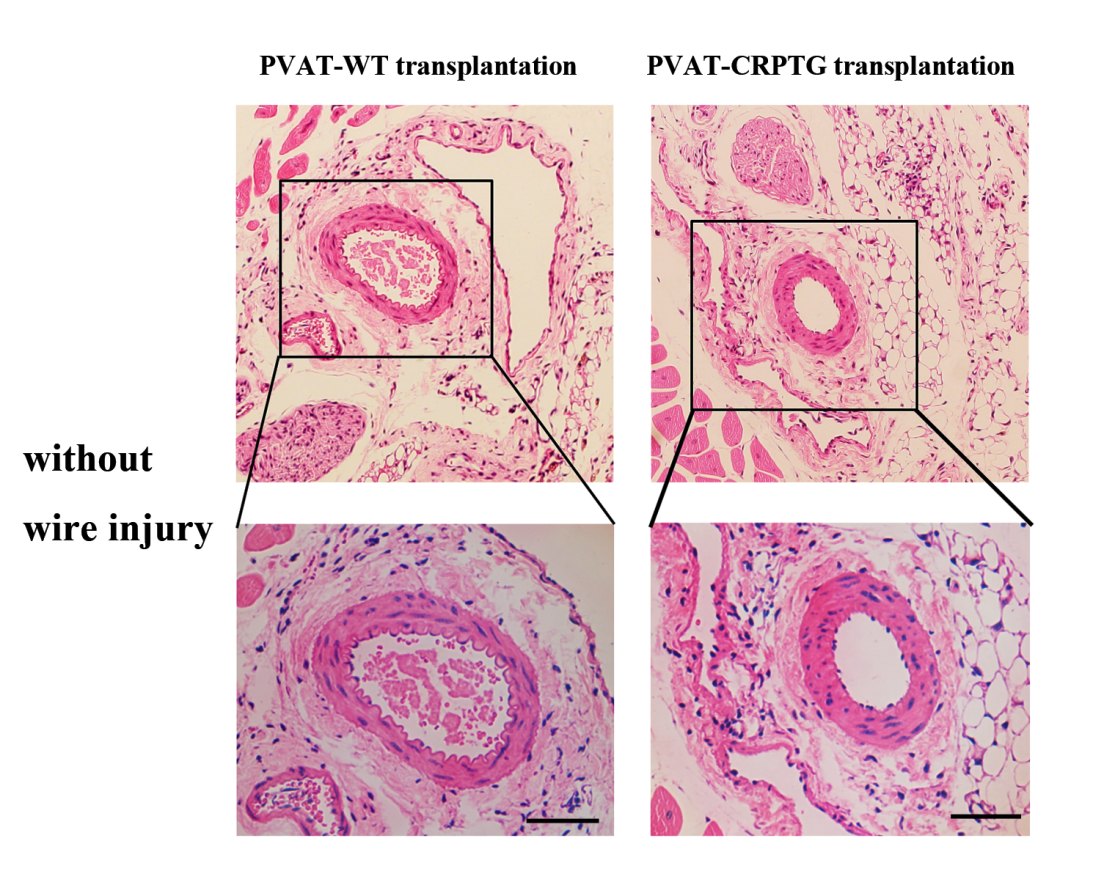


Figure S1. Comparison of neointima thickness in the presence of wild-type and CRPTG PVAT transplantation without wire injury. Scale bars, 100μm


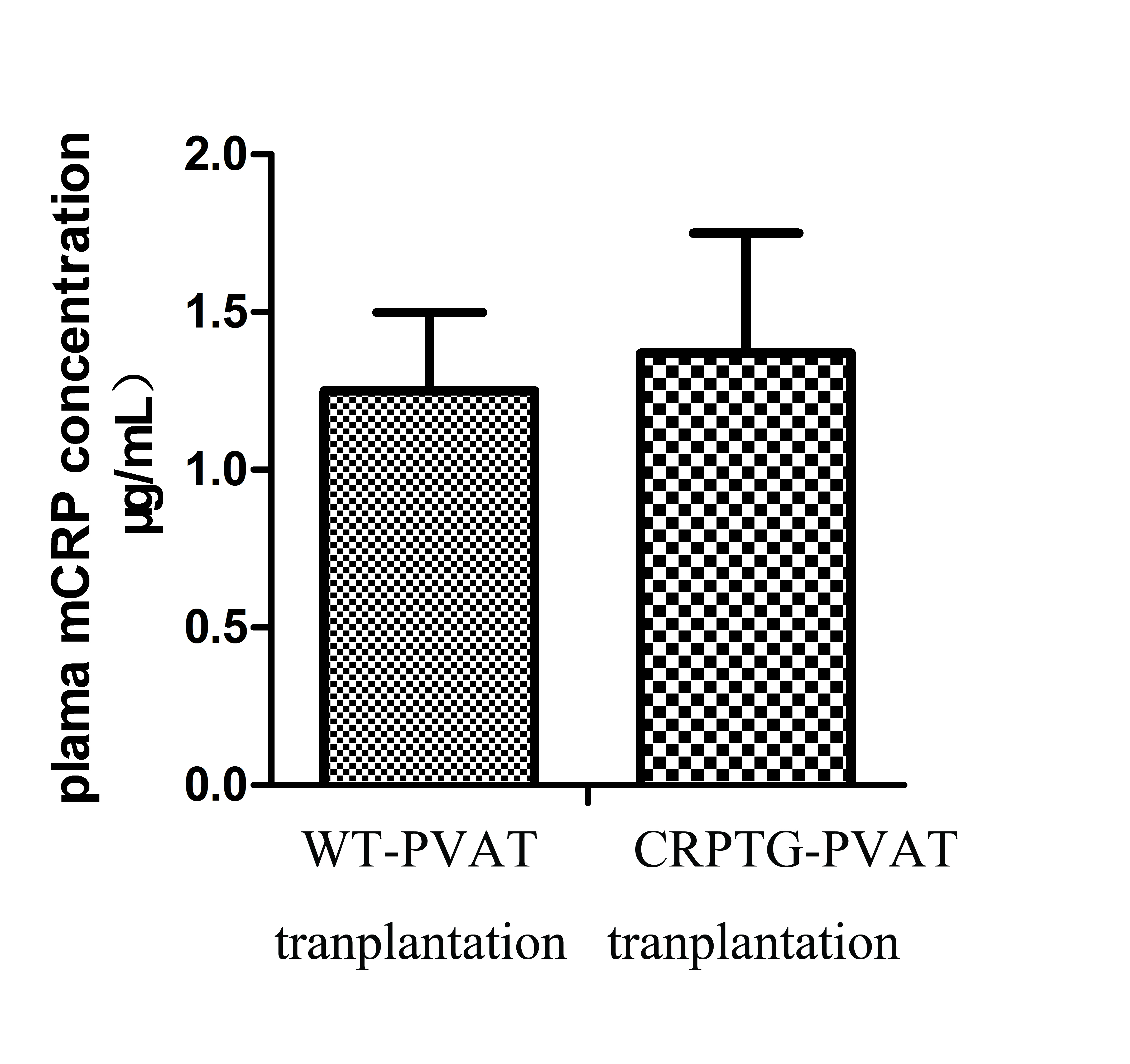


Figure S2. Comparison of plasma mouse C-reactive protein in WT mice received wild-type and CRPTG PVAT transplantation.


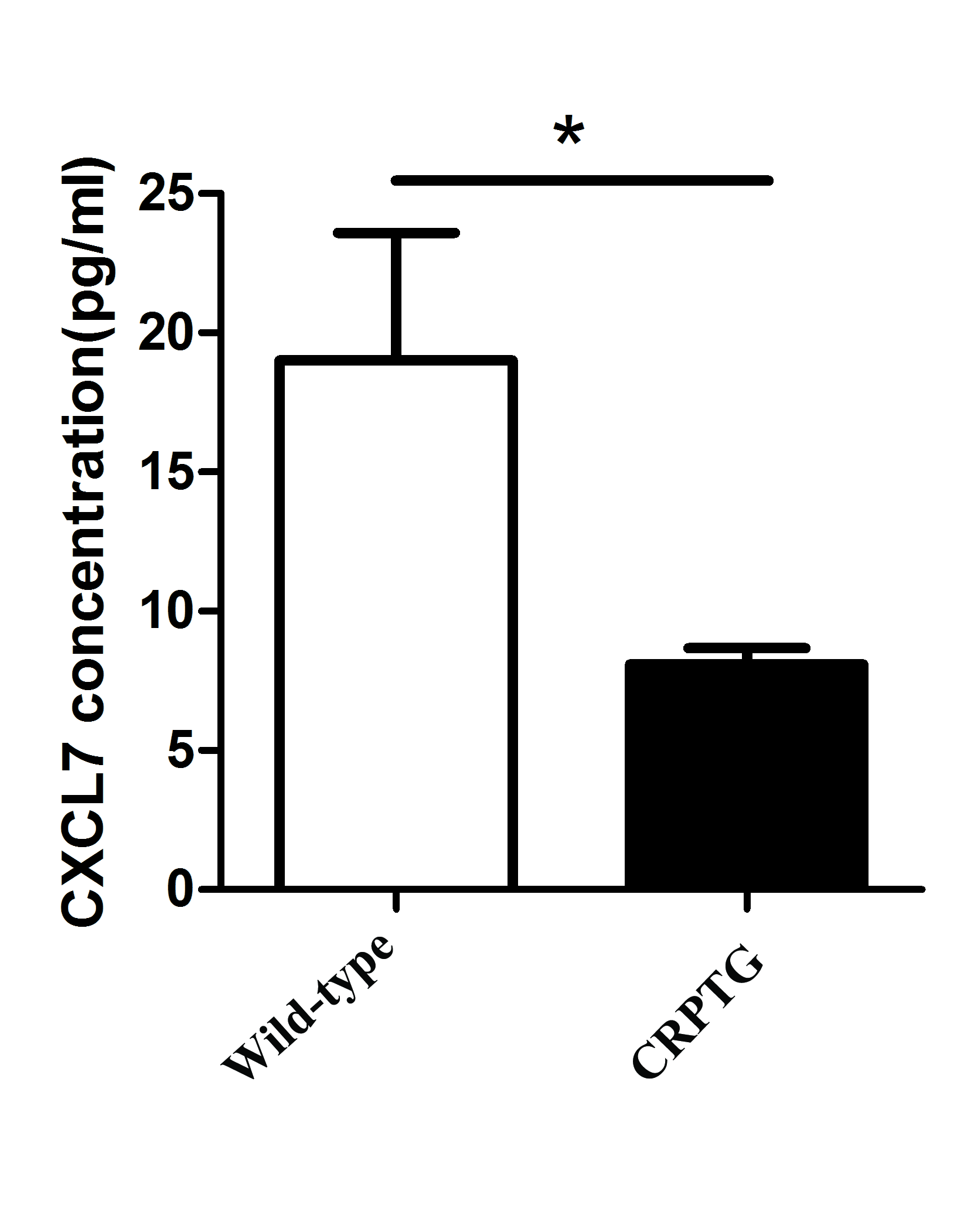


Figure S3. Comparison of plasma mouse CXCL-7 in CRPTG and wild-type mice. *P<0.05 versus wild-type mice
